# Pressurized hot water extraction of hydrosable tannins from *Phyllanthus tenellus* Roxb.

**DOI:** 10.1186/s13065-019-0653-0

**Published:** 2019-12-21

**Authors:** Noor Hidayah Mohd Jusoh, Atiqah Subki, Swee Keong Yeap, Ken Choy Yap, Indu Bala Jaganath

**Affiliations:** 1 Metabolomics Laboratory, Biotechnology Centre, Malaysian Agriculture Research and Development Institute (MARDI), Serdang, Selangor Malaysia; 2 China-ASEAN College of Marine Sciences, Xiamen University Malaysia, Sepang, Selangor Malaysia; 3 Advanced Chemistry Solutions, Damansara Perdana, Petaling Jaya, Selangor Malaysia

**Keywords:** *Phyllanthus*, Phytochemicals, Pressurized hot water extraction

## Abstract

**Background:**

Safety, environmental and economic setbacks are driving industries to find greener approaches to extract bioactive compounds from natural resources. Pressurized hot water extraction (PHWE) is among the solvent free and efficient methods for extracting bioactive compounds.

**Experimental:**

In this study, the suitability of PHWE for extracting bioactive compounds such as phenolics, hydrolysable tannins and flavonoids from *Phyllanthus tenellus* was investigated by UPLC-qTOF-MS.

**Results:**

Solvent properties of water are significantly increased through imposing temperature at 121 °C and pressure at 15 p.s.i. Pressurized hot water extraction obtained 991-folds higher hydrolysable tannins than methanol extraction.

**Conclusion:**

The extraction yields of hydrolysable tannins with PHWE was almost double of absolute methanol extraction.

## Introduction

*Phyllanthus tenellus* Roxb., an herbaceous plant belonging to the family of Euphorbiaceae, is closely related to the commonly known *Phyllanthus niruri*. Similar to *P. niruri*, it is also known for its health benefiting properties. This plant is used to treat urolithiasis, inflammatory bowel disease, diabetes and hepatitis B [1]. *Phyllanthus* sp. are known to contain high levels of hydrolysable tannins which have been frequently associated with its anti-viral activity [2, 3]. Besides, many therapeutic effects have been reported such as anti-oxidant, anti-inflammatory and analgesic effect. Currently there is mounting interest in screening plant sources for anti-viral agents. The genus *Phyllanthus* is also a rich source of phenolics and also contains flavonoids, alkaloids, terpenoids and sterols [4–6].

One of the most important step which determines the final outcome of the quality of the herb is the extraction process. Various organic solvents with different polarities such as methanol, hexane, chloroform, acetonitrile, benzene and ethyl alcohol has been used over the years to optimize extraction of bioactive compounds. However, these solvents are not only toxic both to humans and the environment; they are also expensive especially when carried out at an industrial scale. In more recent studies, extraction has advanced to using new and simpler techniques such as microwave-assisted extraction, supercritical fluid extraction, pressurized liquid extraction and ultrasound-assisted extraction [7, 8].

One of the cleanest and green technologies is just to use water as the solvent. To enhance the solvent properties of water, water in a pressurized hot water extraction (PHWE) can readily alter its physico-chemical properties such as its self-ionization, dielectric constant, viscosity, diffusivity, density and surface tension [9]. PHWE is reported to also exhibit shorter extraction time, lower costs of the extracting agent, and an environmentally compatible technique.

In this study, a system of applying pressure to water at high temperature in an autoclave was used to extract bioactive compounds in *P. tenellus*. We hypothesized that an autoclave treatment could facilitate an efficient extraction of bioactive compounds from *P. tenellus.* This extraction method was compared to water and methanol extraction. The bioactive compounds in the different treatments were profiled using ultra-high pressure liquid chromatography coupled to a quadrupole tandem time of flight (UHPLC-QTOF High Resolution) mass spectrometer system. The results suggest that pressurized hot water extraction can successfully be used for the extraction of *P. tenellus* bioactive compounds as the extraction yields of hydrolysable tannins with PHWE was almost double of absolute methanol extraction.

## Experimental

### Chemicals and standards

The reference standards (gallic acid and ellagic acid) were both purchased from Sigma Chemicals (USA) at purity of 98%. Solvent and mobile phase (acetonitrile, methanol and formic acid) used for LCMS-MS were HPLC grade, which obtained from MERCK, Germany. Ultra-pure water (Aquamax-ultra) was used to prepare all samples and buffer. Pure methanol (100% purity) was used in the preparation of the methanol extracts also obtained from MERCK, Germany.

### Collection and preparation of plant material

Fresh leaves of *P. tenellus* (*MD10525*) were collected from the green house at Malaysian Agricultural Research and Development Institute (MARDI), Malaysia where they were cultivated under semi-controlled environmental conditions. Freshly harvested plants were washed and dried in room temperature. Air-dried leaves were then kept in freezer at temperature of − 80 °C.

### Water extraction at room temperature

About 1 g of chopped air dried leaves of *P. tenellus* were homogenized and thoroughly mixed with 20 mL of double deionized water and then centrifuged at 8900 rpm in 4 °C for 5 min. The supernatant was collected after two rounds of extraction and freeze dried (Labconco, vacuum 0.120 mBAr, temperature − 51 °C). The percentage yield was calculated using the Eq. 1 below;

1 $${{\text{Yield}}}\left( {{\text{Y}}} \right) = \frac{{\text{Wf}}}{{\text{Wi}}} \times 100$$

where W_f_ is the final weight of crude extract (g), W_i_ is initial weight of extract (g). Samples were re-dissolved into final concentration of 20 mg/mL with 30% methanol and filtered through a syringe filter (0.22 μm) before being subjected for UPLC-QTOFF MS system.

### Methanol extraction

About 1 g of chopped air dried leaves of *P. tenellus* were weighed and thoroughly mixed with 20 mL of methanol and then centrifuged at 8900 rpm in 4 °C for 5 min. The supernatant was filtered with Whitman No 1 filter paper and repeated again with adding another 20 mL of methanol. The filtrate was then dried with a vacuum concentrator (Eppendorf concentrator 5301) into powder form. The percentage yield was calculated using Eq. 1 as mentioned before. Samples were re-dissolved into final concentration of 20 mg/mL with 30% methanol and filtered through a syringe filter (0.22 μm) before being subjected for UPLC-QTOFF MS analysis.

### Water extraction at elevated temperatures and pressure

About 1 g of air dried *P. tenellus* leaves were homogenized and thoroughly mixed with 20 mL of double deionize water and then autoclaved for 20 min, at a temperature of 121 °C and a pressure of 100 kPa (15 psi) (Hirayama hiclave, HVE-50). The supernatant was collected and freeze dried (Labconco, vacuum 0.120 mBAr, temperature − 51 °C). The percentage yield was calculated using the Eq. 1. Samples were re-dissolved into a final concentration of 20 mg/mL with 30% methanol and filtered through a syringe filter (0.22 μm) before being injected directly into an ultra-high pressure liquid chromatography coupled to a quadrupole tandem time of flight (UHPLC-QTOF High Resolution) mass spectrometer system.

### Phytochemical detection and identification of *P. tenellus* leaves

Phytochemical analysis of *P. tenellus* was performed using a Eksigent ultraLC 100 UHPLC™ system (Sciex, Framingham, MA, USA), which was equipped with dual pump delivery system coupled to a SCIEX 5600+ QTOF mass spectrometer (Sciex, Framingham, MA, USA) equipped with an electrospray ionization (ESI) interface. Chromatographic separations were performed on a Thermo Hypersil Gold 150 mm × 4.6 mm analytical column. The column was maintained at 40 °C and eluted with a step gradient from 95% acetonitrile (0.05% (v/v) formic acid)—5% aqueous formic acid (0.05% (v/v) formic acid) to 5% aqueous formic acid (0.05% (v/v) formic acid—95% acetonitrile (0.05% (v/v) formic acid) over 67 min at a flow rate of 1.0 mL/min. About 20 μL injection of this sample was made onto the column.

The system was calibrated and optimised using Sciex internal negative ESI tuning mix solutions, which was injected for every three injections between sample runs. The mass spectrometer was operated in negative ion (NI) mode with general unknown screening methodology. An unknown screening mode using TOF MS scan experiment at 100–1200 m/z with information dependant acquisition ion detector array (IDA) of product ion scan (MS/MS) experiment at 50–1200 m/z for fragment ion spectra generation, both experiment performed simultaneously. The pseudo-MS^3^ experiment in the NS/precursor ion MS/MS mode, when performed on a triple quadrupole instrument equipped with an ESI source, involves three steps as described by Chen et al. [10]. 5 maximum number of ions per cycle were monitored, to obtain all relevant ions at proper and acceptable intensity, to be fragmented. The desolvation temperature was set to 500 °C with ionisation and desolvation gas set at 40 psi each. The ion spray voltage was set at 4500 kV with de-clustering potential set at 80 eV.

The data was collected between 100 and 1200 m/z with a low-level collision energy at 10 eV for precursor ion information generation and a collision energy spread of 35 eV ± 15 eV for fragment ion information. Dynamic background subtraction was employed continuously during sample run to reduce background ion interferences. Analyst TF 1.6 software was used for instrument control and data processing. PeakView 2.1 software was used for further data interpretation and fragment ion identifications.

### Calculation of relative quantification for the identified compounds

The relative quantity each constituent detected were calculated as Eq. 2 below;

2 $${\text{Relative quantification }}\left( {\text{Rq}} \right) = \frac{{\text{TIC}}}{{\text{TTIC}}} \times 50$$

where TIC is the total ion count for each compound, TTIC is the total of total ion count for the whole compounds identified in each run and 50 is the dilution factor of the reaction.

## Result and discussion

### Total extraction yield using different solvents

**Fig. 1:**
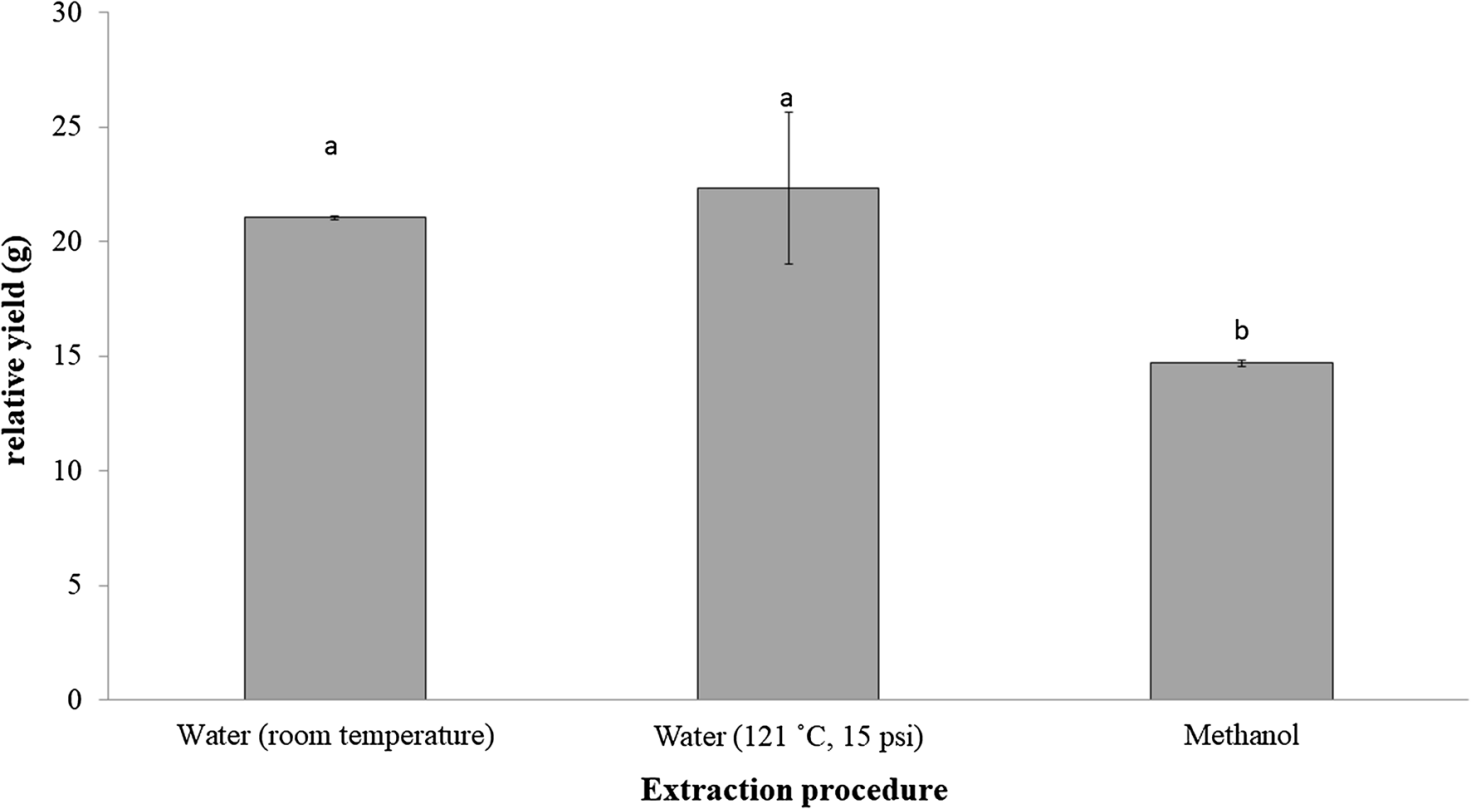
Yield of extraction based on different extraction methods. The yields of different extraction procedures; water (room temperature), water (121 °C, 15 psi) and methanol extract. The order of crude yield: (methanol (14.7) < water (room temperature) (21.05) < water (121 °C, 15 psi) (22.35) extract. Triplicates of results were analyzed by one-way ANOVA with Tukey post hoc and presented as SEM. Different letters indicate significant differences between extract (p < 0.05)

In this experiment, two different solvents were used, water and methanol with three different approaches. Both room temperature and pressurized hot water extract were recorded with significantly higher relative yield than methanol extract (Fig. 1). The PHWE extraction technique was postulated to improve extraction performance by aiding in the rupturing of the cells and release of phytochemicals. In addition, higher temperature increases solubility and simultaneously reduces surface tension of solvents which in turn contributes to higher extraction rate [11]. Previously, the highest extraction yield was also observed in water extract of *P. niruri* Linn by 26.2 ± 1.6 [6] followed by methanol extract at 14.6 ± 1.1. The polarity index by Snyder [12] revealed that water and methanol have polarity index of 9.0 and 6.6 respectively making water a more polar solvent followed by methanol. Methanol as an extraction solvent is usually used for the extraction of semi-polar to non-polar compounds. Water has a dielectric constant of approximately 80 at room temperature. By increasing the pressure and temperature, the dielectric constant decreases and becomes similar to ethanol, indicating that water can be used to extract a wider range of polar and non-polar components [13–16]. This may justify the higher percentage yield when the PHWE was used compared to methanol extract.

### Phytochemical identification and relative quantification

Screening and identification of phytochemicals were achieved by an untargeted UPLC-qTOF-MS at negative ionization mode. For metabolite identification, MS fragmentation patterns were used in conjugation with chemical libraries. Two characteristics wavelengths, i.e. 280 nm for detection of phenolic compounds such as hydrosable tannins and 360 nm for flavonoid [17] were used in this experiment.

A total of 28, 15, and 38 phytochemicals were identified in water (room temperature), PHWE and methanol extract, respectively as shown in Table 1. Different solvents were able to extract different number and/or types of compounds. PHWE can extract from polar to least polar compound while methanol extract semi-polar to non-polar compound. There are four major group of compound that has been extracted, namely, phenolic acids, hydrosable tannins, condensed tannins and flavonoids.

**Table 1:** Identification of chemical constituents in water (121 °C, 15 psi), water (room temperature) and methanol extract of *P. tenellus* leaves

No	Compound identity	m/z	Compound classification	Average relative quantification (%)		
				PHWE	Water (room temperature)	Methanol extract
1	Quinic acid	191	Phenolic acid	0.147 ± 0.050^b^	0.197 ± 0.030^b^	0.068 ± 0.009^a^
2	Caffeoylmalic acid	295	Phenolic acid	0.029 ± 0.008^a^	0.017 ± 0.015^a^	0.361 ± 0.050^b^
3	Caffeic acid 3-glucoside	341	Phenolic acid	0.055 ± 0.040^b^	0.181 ± 0.001^c^	0.007 ± 0.004^a^
4	1-*O*-(2-acetpxybenzoyl)-β-d-glucopyranuronic acid	355	Phenolic acid	0.039 ± 0.013^a^	0.060 ± 0.055^a^	0.030 ± 0.001^a^
5	*o*-coumaroylquinic acid	337	Phenolic acid	0.015 ± 0.002^c^	0.007 ± 0.001^b^	ND^a^
6	Coumaric acid	163	Phenolic acid	0.025 ± 0.002^b^	ND^a^	ND^a^
7	β-glucogallin	331	Phenolic acid	0.149 ± 0.046^c^	ND^a^	0.019 ± 0.007^b^
8	Caffeic acid	179	Phenolic acid	0.024 ± 0.011^c^	ND^a^	0.004 ± 0.004^b^
9	2-*O*-caffeoyl glucarate	371	Phenolic acid	0.063 ± 0.012^b^	ND^a^	ND^a^
10	2,4-Dihydroxy-6-(3-methylbutoxy)-3-(3-methylbutyl) benzaldehyde	293	Phenolic acid	0.007 ± 0.001^b^	ND^a^	ND^a^
11	Chlorogenic acid derivatives	467	Phenolic acid	0.047 ± 0.015^b^	ND^a^	ND^a^
12	Coumaroylquinic acid	337	Phenolic acid	ND^a^	0.017 ± 0.006^b^	0.024 ± 0.001^b^
13	Chlorogenic acid	353	Phenolic acid	ND^a^	0.026 ± 0.074^b^	0.203 ± 0.057^c^
14	2,5-dihyroxycinnamic acid	179	Phenolic acid	ND^a^	0.007 ± 0.002^b^	0.005 ± 0.001^b^
15	1-*O*-(3,4,5-Trihydroxybenzoyl)hexopyranose	331	Phenolic acid	ND^a^	0.005 ± 0.009^b^	0.229 ± 0.017^c^
16	4-*O*-beta-d-glucosyl-4-coumaric acid	325	Phenolic acid	ND^a^	0.007 ± 0.007^b^	ND^a^
17	Rutin	609	Flavonoid	0.039 ± 0.034^b^	ND^a^	0.020 ± 0.014^b^
18	Quercetin glucoside	463	Flavonoid	0.017 ± 0.004^b^	ND^a^	0.254 ± 0.004^c^
19	Myricetin-rhamnoside	463	Flavonoid	0.150 ± 0.005^b^	ND^a^	ND^a^
20	Quercetin glucoside derivative	649	Flavonoid	ND^a^	0.006 ± 0.010^b^	0.110 ± 0.008^c^
21	Cyanidin malonylglucoside	535	Flavonoid	ND^a^	ND^a^	0.013 ± 0.010^b^
22	Cyanidin glucoside	447	Flavonoid	ND^a^	ND^a^	0.005 ± 0.001^b^
23	Kaempferol hexoside	447	Flavonoid	ND^a^	ND^a^	0.061 ± 0.004^b^
24	6-Metoxykaempferol-3-*O*-hexose-*O*-pentose	577	Flavonoid	0.046 ± 0.055^b^	ND^a^	ND^a^
25	Apigenin pentose	401	Flavonoid	ND^a^	ND^a^	0.011 ± 0.009^b^
26	Apigenin	269	Flavonoid	ND^a^	ND^a^	0.002 ± 0.000^b^
27	Naringin	579	Flavonoid	ND^a^	ND^a^	0.039 ± 0.006^b^
28	Catechin	289	Flavonoid	0.004 ± 0.000^b^	ND^a^	0.018 ± 0.003^c^
29	Catechin derivatives	279	Flavonoid	ND^a^	0.003 ± 0.005	0.013 ± 0.004
30	Pelargonidin glucoside	431	Anthocyanin	ND^a^	ND^a^	0.002 ± 0.001^b^
31	Gallic acid	169	Catabolites/metabolites of hydrosable tannin	ND^a^	0.016 ± 0.016^b^	0.030 ± 0.003^b^
32	Gallic acid derivative	521	Catabolites/metabolites of hydrosable tannin	0.034 ± 0.007^b^	ND^a^	ND^a^
33	Gallic acid derivative	537/	Catabolites/metabolites of hydrosable tannin	ND^a^	0.040 ± 0.011^b^	0.761 ± 0.045^c^
34	Ellagic acid	301	Catabolites/metabolites of hydrosable tannin	ND^a^	0.008 ± 0.014^b^	0.124 ± 0.086^c^
35	Ellagic acid glucoside	463	Catabolites/metabolites of hydrosable tannin	0.021 ± 0.009^b^	ND^a^	0.035 ± 0.032^b^
36	Ellagic acid methyl pentoside	447	Catabolites/metabolites of hydrosable tannin	ND^a^	ND^a^	0.025 ± 0.001^b^
37	Methyl ellagic acid hexose	477	Catabolites/metabolites of hydrosable tannin	ND^a^	ND^a^	0.185 ± 0.156^b^
38	Brevifolin	247	Catabolites/metabolites of hydrosable tannin	0.013 ± 0.003^b^	ND^a^	0.010 ± 0.006^b^
39	Brevifolin carboxylic acid	291	Catabolites/metabolites of hydrosable tannin	0.125 ± 0.021^c^	ND^a^	0.011 ± 0.001^b^
40	Brevifolin conjugate		Catabolites/metabolites of hydrosable tannin	0.384 ± 0.029^b^	ND^a^	ND^a^
41	m/z 226	226	Catabolites/metabolites of hydrosable tannin	2.117 ± 3.667^b^	ND^a^	ND^a^
42	m/z 227	227	Catabolites/metabolites of hydrosable tannin	4.679 ± 0.546^b^	ND^a^	ND^a^
43	Geraniin	951	Hydrosable tannin	ND^a^	ND^a^	2.380 ± 0.035^b^
44	Ellagitannin	969	Hydrosable tannin	0.054 ± 0.013^b^	ND^a^	ND^a^
45	Dehydrohexahydroxy diphenic acid	353	Hydrosable tannin	0.067 ± 0.027^b^	ND^a^	ND^a^
46	HHDP-galloyl-glucose	633	Hydrosable tannin	0.407 ± 0.407^b^	ND^a^	ND^a^
47	HHDP glucose	783	Hydrosable tannin	ND^a^	ND^a^	0.008 ± 0.002^b^
48	Corilagin	633	Hydrosable tannin	ND^a^	ND^a^	0.555 ± 0.042^b^
49	Strictinin	633	Hydrosable tannin	ND^a^	ND^a^	0.305 ± 0.147^b^
50	Castalin	631	Hydrosable tannin	ND^a^	ND^a^	0.186 ± 0.261^b^
51	Castalagin	933	Hydrosable tannin	ND^a^	ND^a^	0.458 ± 0.050^b^
52	Proanthocyanidin C1	866	Condensed tannin	ND^a^	ND^a^	0.005 ± 0.000^b^
53	Proanthocyanidin trimer	885	Condensed tannin	ND^a^	ND^a^	0.002 ± 0.002^b^

Triplicates of results were analyzed by one-way ANOVA with Tukey post hoc and presented as SEM. Different letters indicate significant differences between extract (p < 0.05)

*ND* None detected

### Phenolic acids

The phenolic acids and flavonoids that were detected in the extracts are:

Quinic acid (tetrahydroxycyclohexane carboxylic acid) was found in all extracts and the relative quantitative in PHWE was found to be the highest followed by methanol and water (room temperature) extracts. Quinic acid is widely found in plant particularly in coffee bean. The identity of quinic acid with m/z 191, has the characteristic fragments of *m/z* 111 [M−H-80]^−^ (loss of two water (H_2_O) molecules and carbon dioxide (CO_2_), 93 [M−H-18]^−^ (loss of H_2_O) and 87.

B**-**glucogallin is formed from esterification of gallic acid and B-d-glucose. Compound B-glucogallin [M−H]^−^ ion at m/z 331, and MS/MS fragment ions at m/z 211 [M−H-120]^−^. Formation of gallic acid with m/z 169 [M−H-162]^−^ occurred from loss of glucoside and m/z 125 [M−H-44]^−^ (loss of CO_2_). Relative quantification revealed that B-glucogallin is again higher in PHWE extract than methanol extract.

Caffeic acid (3,4-dihydroxy cinnamic acid) with m/z 179 and characteristic MS/MS fragment ion of 135 was found in PHWE and methanol extract. Recent studies emphasized the role of this compound as anticarcinogenic toward skin cancer. 2,5-dihydroxycinnamic acid with m/z 179 and characteristic MS/MS fragment ion of 158, 140 and 135 on the other hand, was found in water (room temperature) and methanol only. Caffeoylmalic acid with m/z 179 and characteristics MS/MS fragment ions of 133,115 and 71 is the ester of caffeic acid was detected the highest in methanol followed by PHWE and water (room temperature) extract. Caffeic acid 3-glucoside with m/z 341 and characteristics MS/MS fragment ions of 179,135 and 133 has been found in water (room temperature), PHWE and methanol tentatively in an increasing order. It is actually a trans-caffeic acid that attached to a beta-d-glucopyranosyl residue at position 3 by glycosidic linkage. Compound 2-*O*-caffeoyl glucarate with m/z 371 and characteristics MS/MS fragment ions of 191, 147 and 85 was identified only in PHWE.

Chlorogenic acids (CGAs) are a type of phenolic compounds formed from the esterification of quinic acid and caffeic acid. Caffeoylquinic acids, coumaroylquinic acid and feruloyl quinic acid are three compounds classes under CGAs. CGAs are abundantly found in coffee bean. Chlorogenic acid (CA) was found in the water extract (room temperature) but not PHWE. This is in line with the nature of this compound, which is thermally unstable. In high temperature, CA will decompose into quinic acid and caffeic acid, which explains the higher presence of these two compounds in the PHWE.

Coumaric acid was found in PHWE only. It has m/z of 163 and characteristics fragments of m/z 119,117 and 93. *o*-coumaroylquinic acid showed a [M−H]^−^ ion at m/z 337, and MS/MS fragment ions at m/z 191 [M−H-146]^−^ (loss of gallic acid) can be found in PHWE. On the other hand, coumaroylquinic acid can be found only in water (room temperature) and methanol extract. Compound 4-*O*-beta-d-glucosyl-4-coumaric acid was identified in water (room temperature) extract only with m/z of 325 and MS/MS fragment ions at m/z 179, 163 and 119.

Other compounds like 1-*O*-(2-acetpxybenzoyl)-β-d-glucopyranuronic acid was found in ascending concentration of water (room temperature), PHWE, and methanol. It has m/z 355 and MS/MS at 211, 169 and 151. Compound 2,4-Dihydroxy-6-(3-methylbutoxy)-3-(3-methylbutyl) benzaldehyde was found only in PHWE extract. It has configuration of m/z of 293 and MS/MS fragment ions at m/z 249, 193 and 136. 1-*O*-(3,4,5-Trihydroxybenzoyl) hexopyranose was found in water (room temperature) and methanol extract only with m/z 331 and fragment ion MS/MS of m/z 168, 149 and 125.

### Flavonoid

Rutin with [M−H]^−^ ion at m/z of 609 and characteristics fragments of m/z with of 301, 300, was identified in PHWE and methanol extract. It is group under flavonoid, made of flavonol quercetin and the disaccharide rutinose. Rutin, also known as vitamin P is an important health benefiting phytochemical and it has been explored for a number of pharmacological effects including possessing cytoprotective, vasoprotective, anticarcinogenic, neuroprotective and cardioprotective activities [18]. Rutin was also identified in *P. niruri* [19].

Quercetin glucoside with [M−H]^−^ ion at m/z 463 and fragmentation ions MS/MS of m/z 316, 271, and 178, was identified in PHWE and methanol extract. Quercetin has many varied health benefits as documented in the review by Kumar et al. [20].

Myricetin-rhamnoside was identified only in PHWE extract with m/z 463 and fragmentation ions MS/MS of m/z 316, 271 and 287. Catechin with [M−H]^−^ ion at m/z 289 and fragmentation ions MS/MS of 109, 121, 125 and 151 was detected in the PHWE and methanol extract.

Kaempferol hexoside with [M−H]^−^ ion at m/z 447 and fragmentation ions MS/MS of 300, 284 and 255 was identified in methanol extract while 6-metoxykaempferol-3-*O*-hexose-*O*-pentose with [M−H]^−^ ion at m/z 577 and fragmentation ions MS/MS of 243, 225 and 125 was discovered only in PHWE.

Other flavonoids such as apigenin, apigenin pentose, naringin cyanidin glucoside, cyanidin malonylglucoside and the anthocyanin pelargonidin glucoside was only detected in the methanol extract.

### Hydrosable tannin

Geraniin is the highest constituent detected and can only be found in the methanol extract. This is in line with the previous studies that revealed geraniin is the most prevalent component in the methanol extract [21]. It has the configuration m/z of 951 and fragment ions of 933, 613 and 301.

Hexahydroxydiphenoyl (HHDP)-galloyl-glucose exhibited a [M−H]^−^ ion at m/z 633, and MS/MS fragment ions at m/z 463 [M−H-170]^−^ (loss of gallic acid), m/z 301 [M−H-332]^−^ (loss of galloyl-glucose) and m/z 275. This compound was only detected in PHWE. HHDP-glucose on the other hand was found only in methanol extract with [M−H]^−^ ion at m/z 783 and fragmentation ions MS/MS of 615 [M−H-168]^−^ (loss of galloyl acid), 597 and 301 [M−H-482]^−^ (loss of HHDP-glucose).

Corilagin was only identified in methanol extract with same [M**−**H]^−^ ion as HHDP-galloyl-glucose at m/z 633, and MS/MS fragment ions at m/z 301, 275 and 249 [M**−**H-146]^−^. Corilagin is the isomer of HHDP-galloyl-glucose [22]. It exhibits activity in lowering the blood pressure through reduction of noradrenaline release. Other than that, previous analysis showed that this compound contains antifungal properties against *Candida glabrata* [23]. The fragmentations of this compound were also comparable with fragmentation in in *Phyllanthus acuminatus* [24].

### Catabolities/metabolites of hydrosable tannins

he hydrosable tannnins have a polyhdric alcohol at their core, the hydroxyl groups of which are partially, or fully, esterified with either gallic acid (in gallotannins) or ellagic acids (ellagitannins). Therefore, upon hydrolysis, they release gallic acid, ellagic acid and their derivatives.

Gallic acid was identified in all extracts. Formation of gallic acid with m/z 169 [M−H-162]^−^ occurred from loss of glucoside and m/z 125 [M−H-44]^−^ (loss of CO_2_), m/z 124 [M−H-45]^−^ (loss of carboxyl ion (–COOH) through decarboxylation) and m/z 79 [M−H-90]^−^. This data is in line with the spectrum of gallic acid in *Nymphaea alba* L. and *Caesalpinia ferrea* [25, 26]. The highest relative quantity was observed from the PHWE followed by methanol and water (room temperature) extraction suggesting the superiority of PHWE method in extracting valuable bioactive compounds. Gallic acid has recently proved to exhibit strong anti-viral properties [27].

Brevifolin was identified in PHWE extract and methanol with [M−H]^−^ ion at m/z 247 and the MS/MS fragment ions at m/z 135,155 and 207. Brevifolin carboxylic acid was identified with [M−H]^−^ ion at m/z 291, and MS/MS fragment ions at m/z 247 [M−H-44]^−^ (loss of carbon dioxide), m/z 191 (galloyl moiety) [M−H-151]^−^ (loss of galloyl group) and m/z 145 [M−H-146]^−^ (loss of coumarin). Brevifolin carboxylic acid has previously proved to exhibit strong cytotoxic activity towards cancer cells [28] and as a potent antiviral herbal drug [29].

Ellagic acid is a dimeric derivative of gallic acid and can be produced from the hydrolysis of ellagitannins. Ellagic acid was predominantly found in methanol extract with (M−H]^−^ at m/z 301 and characteristics fragments MS/MS of m/z 283, m/z 229 [M−H–CO_2_–CO]^−^ and m/z 185 [M−H–2CO_2_–CO]^−^. This finding is in line with the findings of Omar Bakr et al. [25], Zhu et al. [30] and Wyrepkowski et al. [26] where ellagic acid was detected *Duchesnea indica* and *Nymphaea alba*. It possesses antioxidant, antiviral, anticancer, antifibrotic and antimutagenic properties [31]. It can also act a drug-metabolizing enzyme that prevents the formation of toxic metabolite [32]. The more polar ellagic acid glucoside with [M−H]^−^ at m/z 301 was found in PHWE and methanol extract. Ellagic acid methyl pentoside with [M−H]^−^ at m/z 447 and fragmentation ions of 331, 271 and 211 Methyl ellagic acid hexose with [M−H]^−^ at m/z 477 and fragmentation ions of 189, 169 and 137 was only found in methanol extract.

**Fig. 2:**
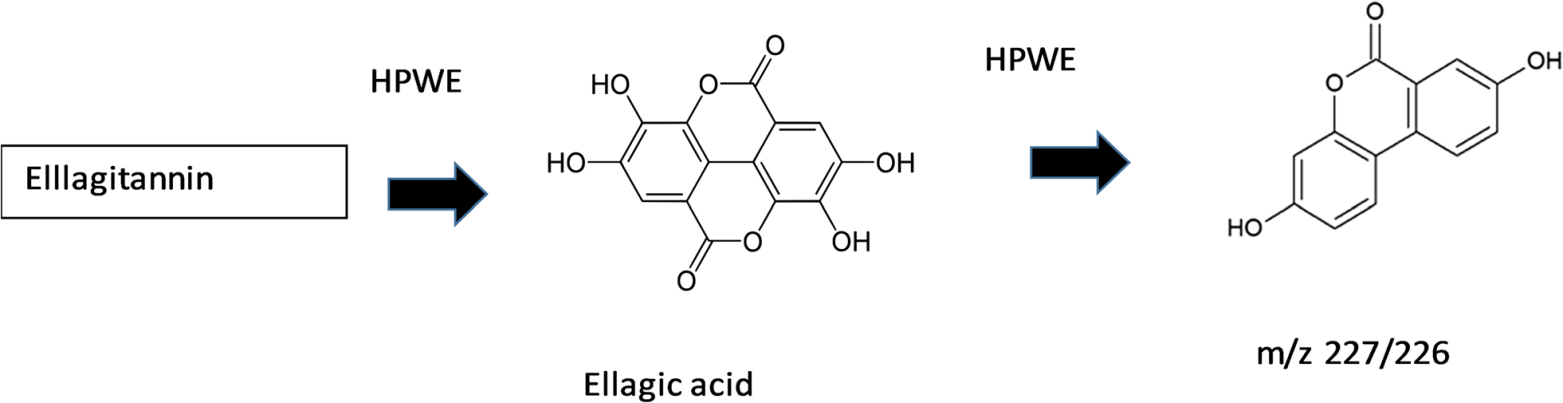
The catabolism of ellagic acid products

Unidentified compounds with mz 226 and mz 227 was detected in abundant quantities in PHWE. It has fragmentations patterns of [M−H]^−^ at m/z 183, 112 and 89. This compound was believed to be a catabolite of ellagitannin/hydrosable tannin known as Urolithin A as indicated in the Fig. 2.

Proanthocyanidin C1 and proanthocyanidin trimer was only identified in methanol extract. Proanthocyanidin C1 has configuration [M−H]^−^ of 866 and MS/MS fragment ions at m/z 695, 577 and 287. Meanwhile, pro-anthocyanidin trimer has m/z 885, and MS/MS fragment ions at m/z 773 and 621.

### Overall comparison of extraction between different classes of compounds

#### Phenolic acids

Caffeoylmalic acid is a hydroxycinnamic acid ester synthesised from caffeic acid and it is moderately non-polar. It dissolves more readily in methanol compared to water as indicated in Table 1. This is in line with the findings of Budzianowski [33] and Cho et al. [34]. Other phenolic compounds such as coumaroylquinic acid, and chlorogenic acid are easily subjected to intramolecular isomerisation, transesterification and degradation with high temperature. These compounds degrade to caffeic and quinic acid [35]. This is evident in this finding where at higher temperature and pressure, significantly higher amounts of caffeic acid, quinic acid are present in the PHWE extract and significantly higher amounts of their precursors, coumaroylquinic acid and chlorogenic acid are present in methanol extract (Table 1). Similar trend is seen in the case of 4-*O*-beta-d-glucosyl-4-coumaric acid, where it degrades to coumaric acid and this is evident by the higher amounts of coumaric acid in the PHWE extraction. β-glucogallin, is formed from gallic acid and β-d-glucose and is the precursor of ellagic acid. Significantly higher levels of β-glucogallin in PHWE extract was obtained compared to the other two extraction methods (Table 1). A plausible explanation would be that the conditions of higher temperature and pressure, could have brought about alterations in the existing structure of the molecule in cells, damaging the plant cell wall, thereby reducing the mass transfer resistance and allowing an easier extraction of phytochemicals such as β –glucogallin and 2-*O*-caffeoyl glucarate [36].

#### Flavonoids

Results from Table 1 and 2 indicate that water at room temperature is a poor solvent for extracting flavonoids from *P. tenellus* unless these compounds are highly polar. The best overall solvent in extracting flavonoids is methanol (Tables 1 and 2). This is in line with the investigations of Dhawan and Gupta [37] and Butsat and Siriamornpun [38]. However, under PHWE conditions, the extraction efficiency of water extraction increase due to its alteration of its self-ionization, dielectric constant, viscosity, diffusivity, density and surface tension as suggested by Khan et al. [36] and Plaza and Turner [39]. With this alteration, water under high temperature and pressure is able to extract significantly higher amounts of flavonoids (42-folds higher) compared to water under room temperature.

**Table 2:** Comparison of compound groups between extracts

Compound group	Total relative quantification (%)		
	PHWE	Water (room temperature)	Methanol
Phenolic acid	0.600^a^	0.580^a^	1.741^b^
Flavonoid	0.256^b^	0.006^a^	0.546^c^
Anthocyanin	0.00^0a^	0.000^a^	0.002^a^
Hydrosable tannin and their metabolites/catabolites	7.933^c^	0.008^a^	4.282^b^
Condensed tannin	0.000^a^	0.000^a^	0.007^a^

Triplicates of results were analyzed by one-way ANOVA with Tukey post hoc and presented as SEM. Different letters indicate significant differences between extract (p < 0.05)

#### Hydrosable tannins

Ellagitannins such as geraniin, strictinin, castalin and castalagin occurs naturally in abundance in *Phyllanthus* sp. and is most soluble in methanol extract. This is apparent by the results obtained these compounds found in relatively large amounts only in methanol extract and none could be detected in water extracts—Table 1). Geraniin, strictinin, castalin and castalagin readily undergo hydrolysis and degradation to its catabolites under heat. Geraninn readily breaks down to brevifolin and its derivatives, corilagin, ellagic acid and gallic acid [40, 41]. With higher levels of heat and pressure, we postulate that corilagin, ellagic acid and gallic acid breaks down further to compounds with mz 226 and mz 227 (Fig. 2). These results are reflected in Table 1 and 2 where hydrosable tannin and their metabolites are present higher amounts in PHWE (1.8-folds higher) compared to methanol extract. In addition, brevifolin and its derivatives were also found to be present almost 25-folds higher in PHWE compared to methanol extract.

## Conclusions

In this study, the effects of pressurized hot water extraction of the *P. tenellus* on the total extraction and component yields were investigated and compared with that of methanol extract and normal water extract. The results revealed that the solvent/polarity properties of water were significantly enhanced and PHWE managed to extract considerably higher amounts of flavonoids (42-folds higher than water at room temperature) and hydrosable tannins together with their catabolites (991-folds higher than water at room temperature). Higher amounts of hydrolysable tannins and their catabolites were recovered in PHWE than the commonly used methanol extract. This is an important finding as pharmacologically active compounds such as brevifolin carboxylic acid, brevifolin derivatives, HHDP-galloyl-glucose were detected in considerably higher amounts compared to normal water extraction and methanol extract. Although the yields of these individual bioactive compounds are improved in PHWE but they are still relatively low in the absolute sense. This however is complemented by the fact that bioactive compounds normally work synergistically together and when combined there are effective even at lower dose. In addition, PHWE does not require any toxic solvents, convenient, safe and environmentally friendly. This is an improvement compared to conventional techniques where large amounts costly solvents are commonly employed. In addition, the step of chopping or macerating leaves together with homogenisation steps are omitted in the PHWE approach, thus saving cost and time when adopted at the industrial scale. Security risks, such as the toxicity of solvents and the presence of solvent residues in the extracts are also omitted when the PHWE approach is adopted. This technique is envisaged to make a substantial difference in adding value to the herbal industry, and will subsequently reduce the cost of the final product.

## Data Availability

The datasets used and/or analysed during the current study are available from the corresponding author on reasonable request.

## Abbreviations

ESI: electrospray ionization
HHDP: hexahydroxydiphenoyl
HPLC: high performance liquid chromatography
IDA: ion detector array
MS/MS: tandem mass spectrometry
MARDI: Malaysian Agricultural Research and Development Institute
NI: negative ion
PHWE: pressurized hot water extraction
TIC: total ion count for each compound
TTIC: total of total ion count for the whole compounds identified in each run
UPLC-qTOF-MS: ultra-high performance liquid chromatography-quadrupole time-of-flight mass spectrometry
W_f_: final weight of crude extract (g)
W_i_: initial weight of extract (g)
